# Risk Factors, Management, and Avoidance of Conduction System Disease after Transcatheter Aortic Valve Replacement

**DOI:** 10.3390/jcm12134405

**Published:** 2023-06-30

**Authors:** Mohamad S. Alabdaljabar, Mackram F. Eleid

**Affiliations:** 1 Department of Internal Medicine, Mayo Clinic, Rochester, MN 55905, USA; alabdaljabar.mohamadsaleh@mayo.edu; 2 Division of Interventional Cardiology, Department of Cardiovascular Medicine, Mayo Clinic, 200 First St. SW, Rochester, MN 55905, USA

**Keywords:** aortic valve stenosis, transcatheter aortic valve replacement, conduction disease, heart block, management

## Abstract

Transcatheter valve replacement (TAVR) is a rapidly developing modality to treat patients with aortic stenosis (AS). Conduction disease post TAVR is one of the most frequent and serious complications experienced by patients. Multiple factors contribute to the risk of conduction disease, including AS and the severity of valve calcification, patients’ pre-existing conditions (i.e., conduction disease, anatomical variations, and short septum) in addition to procedure-related factors (e.g., self-expanding valves, implantation depth, valve-to-annulus ratio, and procedure technique). Detailed evaluation of risk profiles could allow us to better prevent, recognize, and treat this entity. Available evidence on management of conduction disease post TAVR is based on expert opinion and varies widely. Currently, conduction disease in TAVR patients is managed depending on patient risk, with minimal-to-no inpatient/outpatient observation, inpatient monitoring (24–48 h) followed by ambulatory monitoring, or either prolonged inpatient and outpatient monitoring or permanent pacemaker implantation. Herein, we review the incidence and risk factors of TAVR-associated conduction disease and discuss its management.

## 1. Introduction

Aortic stenosis (AS) is one of the three most common heart diseases and the most common valvular disease [1]. The indication for aortic valve replacement (both surgical/transcatheter (TAVR)) is based on symptoms, severity of valvular disease, ejection fraction, and/or the need for other cardiac surgeries. As opposed to surgical intervention, TAVR was initially used to treat AS patients with high/prohibitive surgical risk, but now is a first-line therapy for low-risk patients as well. With TAVR, the most common complications include paravalvular leak, bleeding, acute kidney injury, stroke, and arrhythmia [2]. Of these five, arrhythmia and stroke rates have not decreased substantially [2] with advancement in time, expertise, and devices used. Among arrhythmias, bradyarrhythmia and progressive conduction system disease and their management are the primary focus, owing to the intimate spatial proximity of the aortic annulus with the cardiac conduction system. These include bundle branch block (BBB) and atrioventricular (AV) block of varying degrees.

Many studies have evaluated the relationship between conduction system disease and TAVR, including risk factors, predictors of mortality, and temporal relationships. Herein, we aim to comprehensively review incidence, risk factors, management, and prevention of TAVR-associated conduction disease.

## 2. Incidence and Risk Factors for Advanced Conduction System Disease after TAVR

### 2.1. Incidence of Conduction System Disease with TAVR

The incidence of conduction disease post TAVR has been described by many studies over the years. However, there is a great variability in the reported incidence due to several factors including the focus on various etiologies of conduction system disease (LBBB, high-grade AV block (HAVB), and bradyarrhythmia), time of assessment (early or delayed), and inclusion/exclusion of transient conduction disease, in addition to the differences in demographics and comorbidities of the studied populations, valves used, and other intraprocedural variations. El-Sabawi et al. found that HAVB occurred in 16.1% patients post TAVR, and only 3.7% patients had delayed HAVB (>24 h post TAVR) [3], while 10.5% had an intraprocedural transient HAVB event. Other studies focused on new LBBB post TAVR with incidences ranging between 4 and 65% in the first-generation valves and about 18–65% in the newer generation self-expandable valves [4]. The incidence was higher in other studies including one that assessed conduction abnormalities (LBBB, RBBB, AV block) in 65 patients who underwent TAVR with Medtronic CoreValve system and found that 82% had new conduction abnormality (LBBB most commonly), mainly happening during the procedure [5]. Interestingly, half of the 82% new conduction abnormalities occurred prior to valve deployment. This is a relatively old study wherein the first-generation TAVI system was used, with lower rates of conduction disease being reported when the use of the more recent TAVR systems (i.e., Evolut FX). A comprehensive review article [6] focusing on AV block and permanent pacemaker (PPM) implantation post TAVR included TAVR articles with different study designs and sample sizes ranging between 198 and 42,927 patients. The reported incidence rates of new LBBB ranged between 10 and 39%, while the incidence of PPM implantation ranged widely between 6 and 35%. The majority of the included studies found that there was no association between increased mortality and new LBBB or PPM; however, studies with large sample sizes (>20,000 patients) found an increased mortality risk associated with new LBBB or PPM [7,8,9]. The conduction disease incidence was further broken down based on the type of valve used, with highest rates in mechanically expandable valves (MEV; although a minority of studies included this type of valve), self-expanding valves (SEV), and lowest rates in balloon-expandable valves (BEV) [6]. The main difference between the SEV and BEV is related to the pressure exerted by the valve, wherein the amount of pressure and the rate of deployment are likely higher in the former. As for complete heart block (CHB) specifically, it was estimated that 15% of patients develop heart block requiring PPM post TAVR [10]. These studies show that the wide range of incidence rates requires better defining the population undergoing TAVR to be able to correctly estimate and prevent the risk of conduction disease. The Valve Academic Research Consortium 3 (VARC-3) [11] is an example of a clinical tool that can be used in defining aortic valve clinical research to standardize the definitions used, including conduction disease post TAVR.

### 2.2. Risk Factors for Conduction System Disease

**Figure 1:**
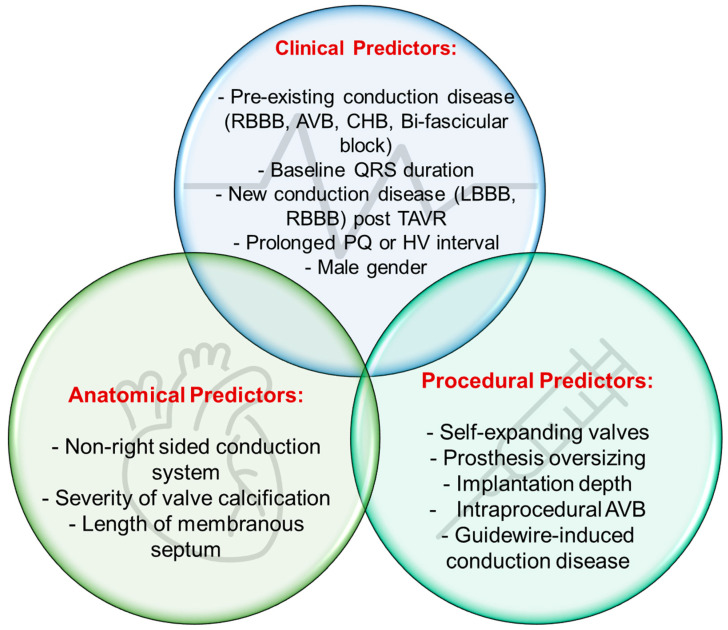
Predictors of conduction disease and/or PPM implantation post TAVR; LBBB/RBBB: Left/right bundle branch block; CHB: Complete heart block; AVB: AV block; TAVR: Transcatheter valve replacement; PQ: the interval between the beginning of P wave to the first ventricular activation (Q).

It is important to understand the proposed mechanism and risk factors behind conduction system disease post TAVR (Figure 1). First, patients with calcific, degenerative age-related severe AS are inherently at a high risk of developing conduction disease regardless of intervention [12,13]. In a study including 1245 patients with varying severity of AS, it was found that around 24% of them had conduction abnormality (LBBB, RBBB, or intraventricular conduction delay), with a proportional relationship between AS severity and conduction disease prevalence [14]. The close proximity between the conduction system (mainly bundle of His and LBB) and aortic valve annulus is an important factor to consider, which makes the LBB more prone to injury with valve deployment (i.e., ischemia, hematoma, inflammation, and/or direct pressure/mechanical injury) [15], however this depends on the anatomical location of the AV node and distal conduction system, and thus, some patients can be at a higher risk to conduction disease due to anatomical variability. One study [16] evaluated the anatomical location of the AV bundle based on autopsy of 115 patients, wherein the majority (50%) had a right-sided bundle, but some had a left-sided bundle (30%) and 20% had a superficial bundle located within the membranous septum (MS). This suggests that patients with non-right sided AV bundle could be more prone for LBBB (or other conduction disease) with TAVR based on their anatomy [17]. However, other studies suggested that anatomical location is not associated with higher risk of conduction disease [18]. Lin et al. [19] have shown in their review paper a graphical illustration highlighting the close proximity between the aortic annulus, membranous septum, and the conduction system (Figure 1).

When it comes to predictors of conduction disease or PPM implantation, it can be thought of as individual-based (clinical and anatomical) and procedural-related factors (Figure 1). These include pre-existing first-degree AV block, RBBB [20], and development of new LBBB [3,18]. Intraoperatively, several risk factors are associated with higher risk of HAVB, including increased oversizing of the implanted prosthesis (e.g., >10% for balloon expandable valve, >15% oversizing with self-expanding valve), aortic annulus stretching, self-expandable valves, and depth of implantation [10]. Oversizing of implantable valve is carried out to ensure valve anchoring and to decrease the risk of valve migration and the risk of paravalvular leak. While these are important considerations, optimal assessment of native valve size and morphology are important to avoid overstretching the aortic valve annulus and/or increase the depth of implantation, both of which are considered risk factors. The access approach used in TAVR between transfemoral versus alternative access is controversial, since some reports showed that the transfemoral approach could increase the risk for PPM [21,22], while others reported no difference [23].

Another proposed risk factor for conduction abnormality and pacemaker dependence is the degree of aortic valve calcification. In a study including 262 patients who underwent balloon-expandable TAVR, 11.1% of patients required PPM implantation. Among those, 68% were pacemaker dependent by 30 days. Factors that were associated with pacemaker dependency at 30 days include non-coronary cusp (NCC) calcium volume (*p* = 0.01), pre-existing RBBB (odds ratio (OR) 105.4, *p* = 0.004), bi-fascicular block (OR 12.5, *p* = 0.02), QRS duration (OR 70.43, *p* = 0.007), and complete heart block (OR 12.83, *p* = 0.03). Moreover, in the NCC group, a calcium volume of >239.2 mm^3^ in the NCC was significantly predictive of pacemaker dependence at 30 days [24]. The length of MS was also found to be an important predictor of conduction disease/PPM implantation rate. Hokken et al. [25] found in their study (653 TAVI patients, 18.4% had PPM implanted) that patients with PPM had a short MS (2.9 mm [IQR 2.3–4.3] versus 4.2 mm [IQR 2.9–5.7], *p* < 0.001). Furthermore, using computed tomography measurement, patients were stratified into risk groups including high risk (MS < 3 mm, 30.3% PPM), intermediate risk (3–6 mm, 15.4% PPM) and low risk group (>6 mm, 6.3% PPM). This was also shown by a recently published meta-analysis [26] including 5740 patients with a higher risk of conduction disease/PPM implantation rate (per 1 mm decrease in MS length, OR = 1.60, 95% CI 1.28–1.99, *p*  <  0.001). Additionally, lower MS and its interaction with implantation depth (ΔMSID) was found to increase the risk of conduction disease/PPM implantation rate (per 1 mm decrease: OR = 1.75, 95% CI 1.32–2.31, *p*  <  0.001). As for PPM implantation, it is important to note that currently many PPM are implanted due to higher risk of developing HAVB prophylactically. In a meta-analysis that included data from 41 reports (11,210 TAVR patients), 17% required PPM implantation, with a range of 2–51% from the studies included. Predictors of PPM implantation included male gender, first degree AV block, left anterior hemiblock, RBBB, and patients with intraprocedural AV block. These results were mainly significant in patients with the Medtronic CoreValve Revalving System, while data for the Edwards SAPIEN valve were limited [27]. This is an important aspect to note, as it is not clear whether this large variation in PPM implantation is due to true high rates of HAVB requiring PPM implantation, due to difference in procedure outcomes in different centers, and/or due to different criteria used to define who would need PPM implantation. Future studies should aim to better define patients who would benefit from PPM implantation in the absence of HAVB post operatively or at the time of discharge.

## 3. Prevention and Detection of Conduction System Disease after TAVR

Similar to any procedure, understanding risk factors and how this could be dealt with is key in prevention. Currently, there are no clear guidelines on how to screen and prevent HAVB, and thus, the rates of PPM implantation vary from institute to another [10]. The 2020 ACC expert consensus provides an approach to evaluate the risk of HAVB block (i.e., third-degree, second-degree type II) in patients undergoing TAVR [10]. Before TAVR, patients should be evaluated for risk factors or underlying rhythm problems. This could help patients with pre-existing conduction disease requiring PPM or could allow further testing in the presence of certain rhythms/risk factors that could be optimized before TAVR, especially since symptoms of underlying rhythm disease and AS could overlap (e.g., pre/syncope). ECG and CT scans are examples of such tests that can help risk stratify patients based on the available data from the literature. ECG could show baseline rhythm issues, including heart blocks, or RBBB [28], while CT scans help in structural evaluation, annulus assessment, and defining the anatomy, although with limitations. Other factors that could be assessed in future studies include medications with AV nodal blockade effects and the optimal time to hold them before TAVR. For instance, in patients undergoing cardiac surgery repair for congenital disease, pre-surgical digoxin exposure (OR 2.4, 95% CI 1.3–4.4) was found to be associated with post operative HAVB [29].

Intraprocedural, type of valve used (i.e., CoreValve), valve to annulus ratio, the position of valve deployment and occurrence/duration of rhythm issues (transient or permeant heart block) [30], or other risk factors reported or identified by the institute should also be considered to mitigate the risks, improve the outcomes, and help decide whether a PPM or post-TAVR rhythm monitoring is required. For example, it is now clearly documented that self-expanding valves have higher rates of conduction disease than balloon expandable. Moreover, valve-to-annulus or prosthesis-to-LV-outflow-tract-diameters ratio [31] is also an important predictor for conduction disease that should be optimized, although studies are lacking on how to best optimize it without compromising procedure success rate (subsequent stenosis, aortic insufficiency, valve migration/embolization). Likewise, Sammour et al. [32] showed that high deployment technique (HDT) reduces the implantation depth, with similar implantation success rates but a better safety profile when it comes to conduction disease (30-day PPM rates with HDT 5.5% versus 13.1%; *p* < 0.001, CHB 3.5% versus 11.2%; *p* < 0.001, and new LBBB 5.3% versus 12.2%; *p* < 0.001). The stiff guidewires used during the TAVR procedure are also culprits for conduction system disease, as it is not unusual to develop new QRS widening after placement of the wire, likely due to the stiff wire hugging the outer curvature of the aorta and thus exerting force on the MS and conduction system.

Post TAVR, the approach is individualized based on pre- and intra-TAVR assessment of conduction system disease. This is done to guide the treating team whether a patient can be safely discharged, requires in-patient or outpatient monitoring (i.e., cardiac monitor), or needs PPM implantation (as prevention or treatment) [10]. In patients at high risk, inpatient monitoring or outpatient ambulatory cardiac monitoring is key in detecting possible HAVB.

Another important entity to consider is early versus delayed (i.e., ≥48 h from TAVR) HAVB. Early is relatively easier to detect, whereas late requires either prolonged hospital stay for monitoring or ambulatory cardiac monitoring on discharge. In high-risk patients with certain risk profiles, PPM implantation is performed prophylactically; however, it is unclear whether all patients who receive PPM prophylactically are in need of one. The current literature lacks studies answering this question with large sample sizes. Delayed HAVB is defined if the conduction disease is detected >48 h or after discharge [10]. This is challenging to diagnose as patients can leave the hospital with no signs/symptoms suggestive of a diseased conduction system, which would put patients at risk of HAVB-related sequalae and complicate objective assessment of the incidence and management of this entity. Delayed HAVB has been estimated to happen in around 10–15% of patients within 30-day post TAVR [10]. A study was carried out to evaluate delayed HAVB in patients undergoing simultaneous TAVR and electrophysiologic testing (*n* = 59). Patients had loop recorder implantation on discharge and were followed for a duration of 12 months. In this cohort the incidence of HAVB was around 12%, and these patients were diagnosed between 2 days and 3 months post TAVR. The prolongation of the PQ (OR: 1.04, 95% CI: 1.01–1.09, *p*: 0.032) and the HV (OR: 1.07, 95% CI: 1.02–1.14, *p*: 0.015) intervals were found to be predictors of delayed HAVB [33]. For the seven patients with delayed HAVB, they underwent PPM implantation with a mean pacing rate of 21.2% (0.5–63.0%) at the 3-month follow up point. In the same study, three patients had transient intra-procedural CHB, and there was no evidence of recurrence post TAVR. Muntané-Carol et al. [34] (*n* = 459) showed that the incidence of post discharge HAVB/CHB was slightly less (4.6%) and was the highest in patients with baseline RBBB and patients with new-onset conduction disturbances post TAVR.

Currently, more novel tools are being developed and are under investigation to better diagnose and reduce TAVR-related conduction disease including the Cara CDRM (Conduction Disturbance Risk Monitor) with a prospective, multicenter, pilot clinical trial (NCT05465655). The hypothesis is that by detailed conduction assessment before, during and after the procedure made possible by this novel monitoring device, steps can be taken to minimize PPM risk and to better predict patients who will benefit from PPM. This study is currently in the recruitment phase with an estimated primary completion date by 31 December 2023. There is growing interest in employing artificial intelligence and machine learning tools to predict the risk of progressive conduction disease after TAVR. One recent analysis [35] demonstrated the ability of machine learning to better predict need for PPM after TAVR compared to a traditional risk score.

## 4. Management of Conduction System Disease after TAVR

**Figure 2:**
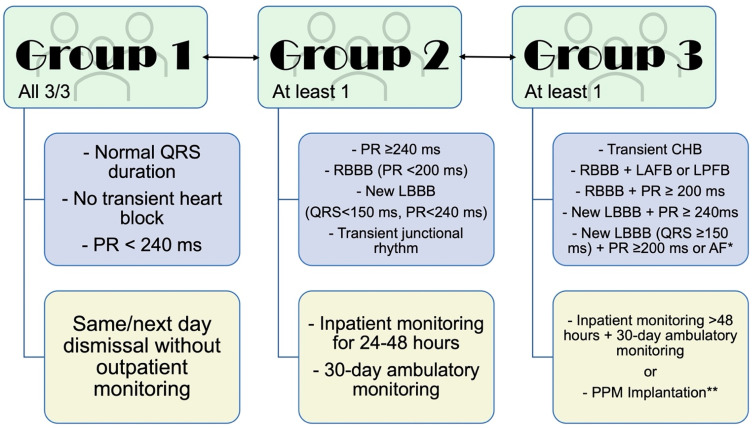
Suggested algorithm for the management of conduction disease post TAVR. * Unmeasurable PR interval due to atrial fibrillation (AF); ** If pre-existing conduction abnormalities are unchanged following TAVR, PPM is based on risks/benefits discussion. LBBB/RBBB: Left/right bundle branch block; CHB: Complete heart block; LAFB/LPFB: Left anterior/posterior fascicular block.

To account for early and delayed potential HAVB, many algorithms have been suggested to approach conduction disease and PPM implantation post TAVR and are mainly institution-specific. Among those, the current approach adopted by the Mayo Clinic, which divides patients into 3 groups, is mainly based on their post-procedure 12-lead ECG (Figure 2); group 1 (normal QRS duration, PR < 240 ms, and no transient HAVB event), group 2 (new LBBB + PR < 240 ms + QRS < 150 ms, isolated PR ≥ 240 ms, isolated RBBB + PR < 200 ms, or transient junctional rhythm); and group 3 (transient HAVB, new LBBB + PR ≥ 240 ms, new LBBB + QRS ≥ 150 ms + PR ≥ 200 ms/incalculable PR, or RBBB with 1st degree AVB/left fascicular block). Management of group 1 includes same-day or next-day discharge with no monitoring, group 2 inpatient monitoring for 24–48 h followed by discharge on 30-day cardiac monitor, while group 3 management includes PPM implantation or prolonged inpatient monitoring with backup temporary pacing and discharge on a heart monitor. This simple and easy-to-follow approach does not account for other clinical/imaging/procedure related features that could make patients more/less prone to permanent HAVB development; however, these categories (clinical/imaging/procedure) could be more challenging to objectively evaluate pre/post TAVR and include in such algorithms due to evaluator variability. Moreover, while patients in group 3 are advised to have PPM implantation, it is unclear whether they would be truly pacemaker-dependent or not, thus, this might overestimate the true number of patients who are in need of PPM implantation. Prolonged hospitalization post TAVR is also a troublesome option since, as evident by other studies, some patients can still develop delayed HAVB up to months after intervention. While these are valid questions on available approaches to conduction disease management post TAVR, these algorithms are on the conservative side to protect patients against potentially serious complications of out-of-hospital HAVB, until more evidence is available to change practice on how to deal with this group of patients. Ultimately, the goals are to reduce the risk and improve screening tools to bridge the gap of conduction disease and conduction-disease-related complications post TAVR.

It is crucial to identify and prevent conduction disease post TAVR not only for the risk of the conduction disease itself, but also because its treatment (i.e., PPM implantation) may negatively influence outcomes post TAVR. In a study [36] of TAVR patients (*n* = 672; 146 had PPM implanted), it was found that PPM patients were at a higher risk for heart failure admissions (HR = 1.70; 95% CI 1.10–2.64; *p* = 0.019) and overall mortality (HR 1.42; 95% CI 0.99–2.05; *p* 0.062). At 1-year follow-up, 30 of 55 (54.5%) patients demonstrated >40% RV pacing. Moreover, in the PPM group, patients with high right ventricular pacing rate >40% were more prone to have heart failure admissions (HR = 5.0; 95% CI 1.23–20.27; *p* 0.007) with increased mortality rate (HR = 2.78; 95% CI 0.86–9.00; *p* 0.064). A subsequent study [37] reported similar findings, with the negative influence of RV pacing ≥ 30% at 1 year (HF readmissions [HR = 6.33; 95% CI 1.417–28.311; *p* = 0.016], all-cause mortality and/or HF [HR = 2.45; 95% CI: 1.040–5.786; *p* = 0.040], atrial fibrillation burden [24.1 ± 40.6% versus 1.2 ± 5.3%; *p* = 0.013], and reduction in ejection fraction [−5.0 ± 9.8% vs. +1.1 ± 7.9%; *p* = 0.005]). Predictors of right ventricular pacing burden ≥30% were found to be valve implantation depth (from non-coronary cusp) ≥4.0 mm (HR = 6.82; 95% CI: 1.829–25.402; *p* = 0.004) and pacing burden of ≥40% at one month (HR = 57.81; 95% CI: 12.489–267.584; *p* < 0.001). These results show the negative effect of pacing on TAVR patients, acutely and on the long-term, with higher burden rate further worsening the outcomes in patients with PPM. Moreover, high pacing burden was associated with the depth of implantation, which appears to be a risk factor for conduction disease, PPM implantation, and also higher pacing burden.

Another point is related to patients at risk without a clear indication for PPM implantation could benefit from temporary pacing. This has been evaluated and the current evidence [38] suggests that this approach is reasonable in this population, although this population should be better defined as we bridge the gap on who should/not receive a PPM. The transient pacemaker inserted during TAVR can also be used for atrial pacing with high negative predictive value to rule out HAVB in that moment. This includes another tool that could help guiding PPM implantation, namely the use of right atrial pacing post TAVR. The concept involves rapid atrial pacing (70–120 beat/min) with assessment for Wenckebach AVB. Krishnaswamy et al. [39] followed this approach reporting a negative predictive value of 98.7% for PPM implantation. Another provocative, yet promising, transient pacing system is the “dissolving” pacemaker [40]. Although it is under development for the time being, this could also be applied in patients at risk of conduction disease post TAVR which could decrease hospitalization rate while provide safety net for any HAVB event. Another concept under investigation is that of combined valve-pacing systems which may allow for interfacing between leadless pacing technology and transcatheter valve technology.

## 5. Future Directions

The management of TAVR-related conduction disease is rapidly developing which is evident by the presence of established approaches to detect, prevent, and treat this entity. The conduction of more studies on bigger scales to evaluate these approaches, and the development of newer tools that could be of benefit to help patients in this group is crucial. When it comes to PPM, other options are available which should be compared, on larger scales and prospective fashion, in TAVR patients requiring pacing. This includes His–Purkinje conduction system pacing (HPCSP) and leadless pacemaker systems (LPS). HPCSP has been shown to be a feasible approach in TAVR patients, albeit more challenging than conventional systems especially in patients with self-expanding as opposed to balloon-expandable valves [41]. HPCSP/His bundle pacing (HBP) could help mitigating risk of dyssynchrony and associated cardiomyopathy. This was shown previously evident by the reduction in heart failure hospitalization rates and pacing-induced cardiomyopathy [42,43]. However, HBP could be technically challenging with a success rate of only 50% in one study [44]. LPS, on the other hand, could reduce the associated major complications, hospitalization, and the need for system revision. This was shown by El-Chami et al. [45]; however, the same study showed that the death rate (0.28% vs. 0%, *p* = 0.0109) and loss of device function (0.50% vs. 0%, *p* = 0.0003) was higher in the LPS (Micra) group at 12 months. The incidence of both death and device function loss was very low, nonetheless. This was also supported by a meta-analysis focusing on LPS (mainly Micra) showing similar results of reduction in major complications and good electrical performance of up to 1 year post implantation [46]. Of note, major complications were defined as events leading to death, readmission, permanent loss of organ/device function, prolonged hospitalization (i.e., 2 days), or intervention/revision, and were compared as a total number between the two groups in both studies. Garweg et al. [47] assessed the safety and efficiency of LPS in patients with valvular interventions (total of 170 patients, 31.8% had valve interventions [16.5% single aortic valve intervention, 5.9% TAVR]). LPS implantation was successful in all patients, and both groups had similar electrical performance and safety profiles. Expectedly, both groups had also a similar reduction in ejection fraction that correlated with the right ventricular pacing rate. Mechulan et al. [48] have shown similar results in a total of 20 TAVR patients with LPS (Micra) implantation. Patients had low complication rates and stable performance at 1-month follow up.

## 6. Conclusions

Management of conduction system in patients undergoing TAVR is one of the most important aspects of TAVR patient management. A comprehensive approach involves identification of patients at increased risk and developing patient-centered individualized approaches to minimize periprocedural morbidity, to prevent the need for a pacemaker whenever possible, and to reduce post-procedural complications and risk of delayed need for PPM. Many ongoing studies, innovative techniques and forthcoming novel technologies promise to further optimize conduction system management in TAVR patients.

## Data Availability

Not applicable.

## Abbreviations

AS	aortic stenosis
AV	atriventricular
BEV	balloon-expandable valves
CHB	complete heart block
HAVB	high-grade AV block
HBP	His bundle pacing
HDT	high deployment technique
HPCSP	His–Purkinje conduction system pacing
LBBB/RBBB	left/right bundle branch block
LPS	leadless pacemaker systems
NCC	non-coronary cusp
MEV	mechanically expandable valve
MS	membranous septum
PPM	permanent pacemaker
SEV	self-expanding valve
TAVR	transcatheter aortic valve replacement
